# Towards a Precision Medicine Approach and In Situ Vaccination against Prostate Cancer by PSMA-Retargeted oHSV

**DOI:** 10.3390/v13102085

**Published:** 2021-10-16

**Authors:** Andrea Vannini, Federico Parenti, Daniela Bressanin, Catia Barboni, Anna Zaghini, Gabriella Campadelli-Fiume, Tatiana Gianni

**Affiliations:** 1 Department of Experimental, Diagnostic and Specialty Medicine, University of Bologna, 40126 Bologna, Italy; andrea.vannini5@unibo.it (A.V.); federico.parenti5@unibo.it (F.P.); daniela.bressanin2@unibo.it (D.B.); 2 Department of Veterinary Medical Sciences, University of Bologna, 40126 Bologna, Italy; catia.barboni@unibo.it (C.B.); anna.zaghini@unibo.it (A.Z.)

**Keywords:** oncolytic virus, oncolytic herpes simplex virus, retargeting, PSMA, prostate cancer, vaccination, in situ vaccine, immunotherapy, immune checkpoint inhibitors

## Abstract

Prostate specific membrane antigen (PSMA) is a specific high frequency cell surface marker of prostate cancers. Theranostic approaches targeting PSMA show no major adverse effects and rule out off-tumor toxicity. A PSMA-retargeted oHSV (R-405) was generated which both infected and was cytotoxic exclusively for PSMA-positive cells, including human prostate cancer LNCaP and 22Rv1 cells, and spared PSMA-negative cells. R-405 in vivo efficacy against LLC1-PSMA and Renca-PSMA tumors consisted of inhibiting primary tumor growth, establishing long-term T immune response, immune heating of the microenvironment, de-repression of the anti-tumor immune phenotype, and sensitization to checkpoint blockade. The in situ vaccination protected from distant challenge tumors, both PSMA-positive and PSMA-negative, implying that it was addressed also to LLC1 tumor antigens. PSMA-retargeted oHSVs are a precision medicine tool worth being additionally investigated in the immunotherapeutic and in situ vaccination landscape against prostate cancers.

## 1. Introduction

Prostate cancer (PC) is a frequent cause of cancer among men worldwide, second only to lung tumors, and is the fifth-leading cause of cancer-related death in men [1,2]; it is the most common cancer among men in 84 countries. In 2018, it was diagnosed in 1.2 million patients and caused 359,000 deaths. At diagnosis, prostate cancers fall into three basic groups [3]. In the first group, the tumors present as localized; patients undergo active surveillance, and a fraction of them progress to overt disease. The second group (1/3 of all new cases) includes locally advanced tumors; patients are at significant risk of disease progression and cancer-related death. In the third group, the disease is metastatic at diagnosis. The majority of patients from the second and third groups progress after androgen deprivation (castration) treatment. Prostate cancers are rather slow growing tumors, usually moderately sensitive to immune check-point inhibitors (ICIs) [4] and these attributes made PC a good candidate for dendritic cell (DC)-based immunotherapy. The DC-based autologous vaccine called Sipuleucel-T is an immunotherapeutic approach, which elicits an immune response targeted against castration-resistant prostatic acid phosphatase [5]. It is a personalized treatment made from the patient’s blood which works by reprogramming each patient’s immune system. Hence, Sipuleucel-T is not available to a large percentage of patients. Therefore, the need for novel immune therapies in prostate cancer remains a high priority [6], and an off-the-shelf derivative would be advantageous. In particular, there is a need for local control of PC, a need to treat any microscopic and confirmed metastases, and, above all, to vaccinate patients against PC, including those undergoing a watchful waiting protocol [6].

Oncolytic viruses (OVs) or onco-immunotherapeutic viruses (OIVs) are promising candidate anticancer therapeutics in the preclinical and clinical stages of development [7,8]. OVs exploit engineered or natural human viruses which are partly devoid of pathogenicity—hence, they are partially attenuated—but retain the ability to infect, replicate in, and destroy cancer cells selectively. As an alternative, OVs consist of animal viruses capable of infecting and replicating selectively in human tumor cells. OVs induce modifications to the tumor microenvironment (TME), including the infiltration and activation of lymphocytes, and the secretion of immunomodulatory cytokines which confer or increase sensitivity to ICIs and, therefore, OVs are considered to be the ideal partners for ICIs [9]. An important platform consists of oncolytic herpes simplex viruses (oHSVs), among which is Onco-Vex_GM-CSF_, approved against metastatic cutaneous melanoma [10,11,12]. Oncolytic virotherapy has been considered against PC. The oncolytic viruses, the efficacy of which has been or is being tested in clinical trials against PC, include oncolytic Adenoviruses armed with Interleukin-12 (IL-12) or granulocyte macrophage colony-stimulating factor (GM-CSF), and Reolysin (natural human virus), also in combination with ICIs [13], (see for examples Clinicaltrials.gov reference NCT02555397, NCT02555397, NCT03514836, NCT01619813).

Our laboratory is committed to the construction of cancer-specific oHSVs retargeted to cancer-specific receptors of choice, and detargeted from the natural receptors Nectin1 and HVEM [14,15,16,17,18,19,20,21,22,23,24,25]. Briefly, in vitro and in mice, the retargeted oHSVs do not infect any cells other than the tumor cells which express the targeted receptor; the specific tropism results in a high safety profile in mice (see [24]). The retargeted oHSVs carry no deletion or mutation in virulence genes, or in genes which reduce the virus ability to contrast the antiviral innate response; hence, they are fully virulent within the infected tumor cells, and fully competent in dealing with the innate and adaptive responses [14,20,24,26,27]. The three main features exhibited by the HER2 (human epithelial growth factor receptor 2)-retargeted IL-12-armed oHSVs (our prototype viruses) are as follows [24,28]. They are effective against primary tumors, they induce an adaptive long-term immune response which protects mice from distant challenge tumors (abscopal effect), i.e., they elicit a powerful anti-tumor in situ antigen agnostic vaccination; they synergize with ICIs.

Prostate specific membrane antigen (PSMA) is a tumor-associated antigen (TAA) highly expressed in prostate adenocarcinoma cells regardless of androgen status [29], in the neovasculature of solid tumors [30], and little expressed in benign and extraprostatic tissues. Within the PC cells, the PSMA begins to be upregulated and to migrate to the plasma membrane during the transition to androgen independence, and is, for the most part, associated with high grade, metastatic disease. High PSMA expression is frequent (75%) in metastatic castration-resistant PC patients [31,32,33]. The PSMA is currently utilized for diagnostic or combined diagnostic + therapeutic (theranostics) applications, including PSMA-SPECT (single photon emission computerized tomography) and the delivery of radiometals. The Clinicaltrials.gov site lists 97 trials, the vast majority of which utilize PSMA as diagnostic or theranostic. Notably, the data available clearly indicate that targeting PSMA results in low toxicity, and little off-tumor and off-target effects [34,35]. 

We recently engineered an oHSV retargeted to PSMA, which was capable of infecting and in vitro killing PSMA-positive cells. The objective of this study was to evaluate the in vitro susceptibility of human PC cells to the PSMA-retargeted oHSV, called R-405, to provide the first proof-of-principle evidence that R-405 exerts antitumor efficacy and long-term protection in vivo against PSMA-positive tumors in syngeneic murine models, and to determine whether the treatment elicited a specific adaptive immune response and in situ vaccination against distant challenge tumors.

## 2. Materials and Methods

### 2.1. Cells and Antibodies

PC3 (human prostatic adenocarcinoma), LNCaP (human prostate adenocarcinoma), 22Rv1 (human prostate carcinoma), SK-OV-3 (ovary adenocarcinoma), LLC1 (Lewis Lung carcinoma), and Renca cells (renal adenocarcinoma) were purchased from ATCC (Manassas, VA, USA) and cultured as indicated by ATCC. J cells, their derivatives expressing Nectin1, HVEM and PSMA [36,37] and PC3-PIP cells, which are isogenic to PC3 and stably express PSMA [38] were grown in DMEM (Gibco - Thermo Fisher Scientific, Waltham, WA, USA) supplemented with 5% fetal bovine serum (FBS). The monoclonal antibody (MAb) D2B directed against PSMA was generously provided by Dr. Marco Colombatti and Dr. Giulio Fracasso (University of Verona, Verona, Italy) [39]. Neutralizing MAb 52S to HSV-1 gH/gL and MAb BD80 to HSV-1 gD were described [21,40].

### 2.2. Viruses

The R-LM5 virus was described [15]. The HSV-1-derivative R-405 virus was obtained from BAC R-337 [24] by substitution of the scFv to HER2 with the sequence encoding the J591 scFv directed against PSMA [41]. The scFv substitution was performed by galK recombineering [42] [43]. Briefly, the galK cassette with homology arms to gD was amplified with primers gD37GalKFor ACCTTCCGGTCCTGGACCAGCTGACCCCTCCGGGGGTCCGGCGCGTGCCTGTTGACAATTAATCATCGGCA and gD39GalKRev ATCGGGAGGCTGGGGGGCTGGAACGGGTCTGGTAGGCCCGCCTGGATGTGTCAGCACTGTCCTGCTCCTT and using pGalK as template. Recombinants were checked by PCR with primer galK_129_For

ACAATCTCTGTTTGCCAACGCATTTGG and galK_417_Rev CATTGCCGCTGATCACCATGTCCACGC. The J591 cassette with homology arms to gD was amplified with primers gD37_J59For ACCTTCCGGTCCTGGACCAGCTGACCCCTCCGGGGGTCCGGCGCGTGGAGGTGCAGCTGCAGCAGTCAGGACC and gD39_J591Rev ATCGGGAGGCTGGGGGGCTGGAACGGGTCTGGTAGGCCCGCCTGGATGTGGGATCCACCGGAACCAGAGCCACCGC using BAC R-593 as template [37]. Recombinants were checked by PCR with primers J591_117-141_For GAGCCATGGAAAGAGCCTTGAGTGG and J591_503_527_Rev AGTTTAGGAGATTGTCCTGGTTTCT. After reconstitution of R-405 virus in SK-OV-3-PSMA cells, single plaque purification was performed [42,43]. Virus R-405 expresses Cre-recombinase which excides BAC sequences. To check the loss of BAC sequences, R-405 viral DNA (NucleoSpin Tissue, Macherey Nagel, Duren, Germany) and BAC R-405 were used as templates for RT-PCR with primers specific for BAC sequences (BACRTForw2 GCACATCAGCAGCACATACG and BACRTRev2 GAAGGTAAACTGCCCACCGA) and HSV-1 gD (gDRTFor ACGTCCGGAAACAACCCTAC and gDRTR ATGGGGATAGCACAGTTGCC). Signal of BAC sequences normalized on gD and compared between viral DNA and BAC confirmed the loss of BAC sequences in more than 99.99% of virions. R-405 virus was cultivated in SK-OV-3-PSMA cells.

### 2.3. Generation of Murine and Human Cancer Cells Expressing Human-PSMA

SK-OV-3, LLC1 and Renca cells were made transgenic for the expression of a truncated form of human PSMA, in which the 4 N-terminal amino acids (WNLL) of the human-PSMA gene were deleted. The pIRES-PSMA plasmid [37] was amplified with primers PSMAEcoRVFor GCTCGCGCCGAGATGTGATATCTCATGCACGAAACCGACTCGGCTGTG and PSMAXhoIRev CAAATTCAATACGGATTCTCGAGAGAATCCTCTTAGGC. The PCR fragment digested EcoRV and XhoI was cloned in the lentiviral expression vector pLV-EF1-MCS-SV40-Puro, obtaining pLV-PSMA-puro. SK-OV-3, LLC1 and Renca cells were transduced as detailed [20]. Transduced cells were selected by means of puromycin and single cell clones were obtained by limiting dilution. Clones were checked for stable PSMA expression for up to 40 passages in cell culture by flow cytometry (BD Accuri, BD Biosciences, San Jose, CA, USA) with MAb D2B to PSMA.

### 2.4. R-405 Tropism and Neutralization of Infection by MAbs to PSMA and HSV-1

J cell derivatives, PSMA-positive (SK-OV-3-PSMA, LLC1-PSMA, Renca-PSMA, LNCaP, PC3-PIP, 22Rv1) and PSMA-negative cancer cells (SK-OV-3, LLC1, Renca, PC3) were infected with R-405 virus at an input multiplicity of infection of 10 PFU/cell as titrated in SK-OV-3-PSMA, for 90 min at 37 °C. Pictures were taken 24 h after infection by Nikon Eclipse TS100 fluorescence microscope. For the neutralization assay, replicate monolayers of the PSMA-positive (SK-OV-3-PSMA, LNCaP, PC3-PIP, 22Rv1) and PSMA-negative cancer cells (SK-OV-3) seeded in 96-well plates were preincubated with increasing amounts of MAb D2B or with mouse IgG for 60 min at 37 °C. R-405 (3 PFU/cell) was added to the MAb-containing medium for additional 90 min. Alternatively, R-405 and R-LM5 virions (3 PFU/cell) were pre-incubated with increasing amounts of HSV-1-neutralizing MAb 52S for 1 h at 37 °C, and then allowed to adsorb to cells for 90 min (R-405 to PSMA-positive cancer cells, R-LM5 to SK-OV-3). Viral inoculum was removed, and cells were overlaid with medium containing MAbs for 18 h. The extent of infection was measured by FACS as the percentage of EGFP-positive infected cells. Western blot assay was performed as described [44].

### 2.5. Virus Growth and Cytotoxicity Assay

The number of encapsidated/enveloped infectious and non-infectious viral particles in R-405 stocks was determined as described [42] and expressed as genome copies per ml (gc/mL). R-405 was titrated in the indicated cell lines; plaque number and size were scored after 5 days. The number of viral particles required to obtain a single plaque in the indicated cell lines was calculated as the ratio of gc/mL to the titer values. To measure the extent of virus replication, PSMA-positive (SK-OV-3-PSMA, LLC1-PSMA, Renca-PSMA, LNCaP, PC3-PIP, 22Rv1) and PSMA-negative cancer cells (SK-OV-3, LLC1) were infected at an input multiplicity of 0.1 PFU/cell, as titrated in the corresponding cell line. Unabsorbed virus was inactivated by means of acidic wash (40 mM citric acid, 10 mM KCl, 135 mM NaCl, pH 3.0). Replicate cultures were frozen at the indicated times after infection. The progeny was titrated in SK-OV-3-PSMA cells and plaques were scored 5 days later. For the cytotoxicity assay, PSMA-positive (SK-OV-3-PSMA, LLC1-PSMA, Renca-PSMA, LNCaP, PC3-PIP, 22Rv1) and PSMA-negative cancer cells (SK-OV-3) were seeded in 96 well plates 8 × 10^3^ cells/well and infected with R-405 or mock-infected. The input multiplicity of infection was 0.05 PFU/cell as titrated in the corresponding cell line. AlamarBlue (Life Technologies-Thermo Fisher Scientific, Waltham, MA, USA) was added to the culture media (10 μL/well) at the indicated times after infection and incubated for 4 h at 37 °C. Plates were read at 560 nm and 600 nm with GloMax Discover System (Promega Corporation, Madison, WI, USA). For each time point, cell viability was expressed as the percentage of AlamarBlue signal reduction in infected versus uninfected cells, after subtraction of the background value (medium alone).

### 2.6. In Vivo Experiments

C57BL/6 and BALB/c mice were obtained from Charles River Laboratories (Wilmington, NC, USA) and bred in the facility of the Department of Veterinary Medical Sciences, University of Bologna (Bologna, Italy). Both female and male mice were randomly used throughout experiments, according to a request from the Ethical Committee. Retrospective evaluation of results based on sex-based stratification of mice showed no sex-related difference. LLC1-PSMA or Renca-PSMA cells were implanted subcutaneously in the left flank of 7–12-week old C57BL/6 and BALB/c mice, respectively, in 100 μL of serum-free medium, 1 × 10^6^ cells/mouse. Where indicated, mice were subcutaneously engrafted with LLC1-WT or Renca-WT cells in the right flank, in 100 μL of serum-free medium, 1 × 10^6^ cells/mouse (challenge tumors). Tumor volumes were scored 3 times weekly by measuring the largest and the smallest diameter by means of a caliper. Tumor volume was calculated using the formula: largest diameter × (smallest diameter)^2^ × 0.5. Mice were sacrificed within one or two days after their tumors reached a volume of about 1500 mm^3^, ulceration occurred, or animals exhibited distress or pain. For virus treatment, when the tumor volumes averaged 70–100 mm^3^ (7–9 days post tumor engraftment for LLC1-PSMA cells, 15–16 days post tumor engraftment for Renca-PSMA cells), mice received 2–4 intratumoral injections of R-405 (doses of 0.02 × 10^7^, 0.1 × 10^7^, 0.2 × 10^7^, 0.3 × 10^7^, 0.5 × 10^7^, 1.7 × 10^7^, 3 × 10^7^, 5 × 10^7^ or 1 × 10^8^ PFU–depending on the experiment-per injection in 50 µL of PBS) or vehicle (50 µL PBS), at 2–7 days intervals. Mice which survived the primary tumor were engrafted with challenge tumors: C57BL/6 mice were challenged with LLC1-PSMA (left flank) and LLC1-WT (right flank), BALB/c mice were challenged with Renca-PSMA (left flank) and Renca-WT (right flank). The challenge tumors were not treated. Where indicated, mice received 4 intra peritoneal (i.p.) injections of anti-mouse CTLA4 antibody (200 µg/mouse, clone 9H19, BioXcell, Lebanon, PA, USA) or anti-mousePD-1 antibody (200 µg/mouse, clone RMP1-14, BioXcell), or vehicle (50 µL PBS) at 3–4 days intervals. 

### 2.7. Determination of Tumor Microenvironment Infiltrating Cells

Single cell suspensions were prepared from freshly isolated LLC1-PSMA tumors and spleens at sacrifice. Tumors were minced in small pieces and digested with collagenase (1 mg/mL) for 30 min at 37 °C. The resulting cell suspensions were passed through 70 μm cell strainer and rinsed with flow cytometry buffer (PBS + 2% FBS). Spleens were processed directly through the cell strainer, and the red blood cells in splenic specimens were lysed by means of ACK buffer (150 mM NH_4_Cl, 10 mM NaHCO_3_, 1 mM EDTA), samples were pelleted and resuspended in flow cytometry buffer. For each sample, 2 × 10^6^ cells were blocked with α-CD16/32 Ab (5 µg/mL; clone 93, eBioscience-Thermo Fisher Scientific, Waltham, MA, USA), and then reacted with the antibodies CD45-FITC (5 µg/mL; clone 30-F11, eBioscience), CD45-PE-Cy7 (1.25 µg/mL; clone 30-F11, eBioscience), CD4-FITC (2.5 µg/mL; clone GK1.5, eBioscience), CD8a-PE (1 µg/mL; clone 53–6.7, eBioscience), CD335-PE (10 µg/mL; clone 29A1.4, eBioscience), CD11b-FITC (6.25 µg/mL; clone M1/70, eBioscience), MHC-I(H-2Kb)-APC (2 µg/mL; AF6-88.5.5.3, eBioscience), FoxP3-PE (2.5 µg/mL; clone 150d/e4, eBioscience), IFNγ-APC (1 µg/mL; clone XMG1.2, eBioscience), and CD69-PercP (2.5 µg/mL; clone H1-2F3, eBioscience). To detect Human PSMA, samples were reacted with MAb D2B (10 µg/mL), washed twice, then reacted with Alexa Fluor™ 488 anti-mouse IgG H+L (10 µg/mL; Invitrogen-Thermo Fisher Scientific, Waltham, MA, USA). Staining reactions were performed on ice for 30 min. Data were acquired on BD C6 Accuri. Only samples which provided at least 100,000 events were included in the analysis.

### 2.8. Determination of Intratumoral Cytokine and Immune Factors by RT-PCR

A few mgs of the LLC1-PSMA tumor homogenates were employed for RNA purification with the Nucleospin RNA kit (Macherey-Nagel), including the on-column DNaseI treatment. Two μg of total RNA was employed for the cDNA synthesis using the High-Capacity cDNA Reverse Transcription Kit (Applied Biosystems-Thermo Fisher Scientific, Waltham, MA, USA) in a 20 μL reaction. qRT-PCR reactions were performed in a StepOnePlus System (Applied Biosystems) using 0.5 μL of cDNA for each assay, and the TaqMan probes employed for the assay are: mm00434169_m1 (*Il12a*), mm01168134_m1 (*Ifnγ*), mm00444662_m1 (*Cxcl11*), mm00443258_m1 (*Tnfα*), mm00475162_m1 (*FoxP3*), mm00450960_m1 (*Tbx21/T-bet*), Mm03048248_m1 (*Pd-l1*), *gC* of HSV-1 (Forw ACCTTCACCTGCCAGATGAC, Rev ACATGCCGGACCCCAAATTC, probe FAM™-CCAGGGCCAGCCCGGTGGCA -TAMRA™, [20], mm01612987_g1 (*Rpl13a*). The levels of expression were determined using the ΔΔCt method, normalized relative to the *Rpl13a* housekeeping gene.

### 2.9. Detection of Splenocyte Reactivity to Cancer Cells

Freshly explanted spleens were smashed through a 70 μm cell strainer in PBS to isolate splenocytes. Red blood cells in spleens were lysed with ACK buffer, while the splenocytes were resuspended in medium (RPMI 1640 containing 10% heat inactivated FBS, 1% penicillin/streptomycin), counted and seeded in 24 well plates. Suspensions of LLC1-WT and LLC1-PSMA cells were treated with mitomycin 15 μg/mL (Sigma-Aldrich, St. Louis, MO, USA) for 2 h at 37 °C, then washed 3 times with fresh medium. Splenocytes (1 × 10^6^ cell/well) were incubated either with 1 × 10^5^ LLC1-WT or LLC1-PSMA cells in 0.5 mL medium, and cocultured for 72 h. Media were collected and the amount of secreted IFNγ was quantified by ELISA (IFN-gamma Mouse ELISA Kit, Thermo Fisher Scientific, Waltham, MA, USA).

### 2.10. Detection of Serum Antibodies to Cancer Cells

To detect the serum antibodies to cancer cells, LLC1-WT and LLC1-PSMA cells were trypsinized and resuspended in flow cytometry buffer. For each sample, 0.25 × 10^6^ cells were reacted with mouse serum, diluted 1:150, in 96 well plate in ice for 1 h, rinsed with flow cytometry buffer, and finally incubated with anti-mouse APC (1:200, eBioscience). Data were acquired on BD C6 Accuri.

## 3. Results

### 3.1. Genetic Engineering of R-405 and Generation of Human and Murine Cell Lines Expressing Human PSMA

**Figure 1:**
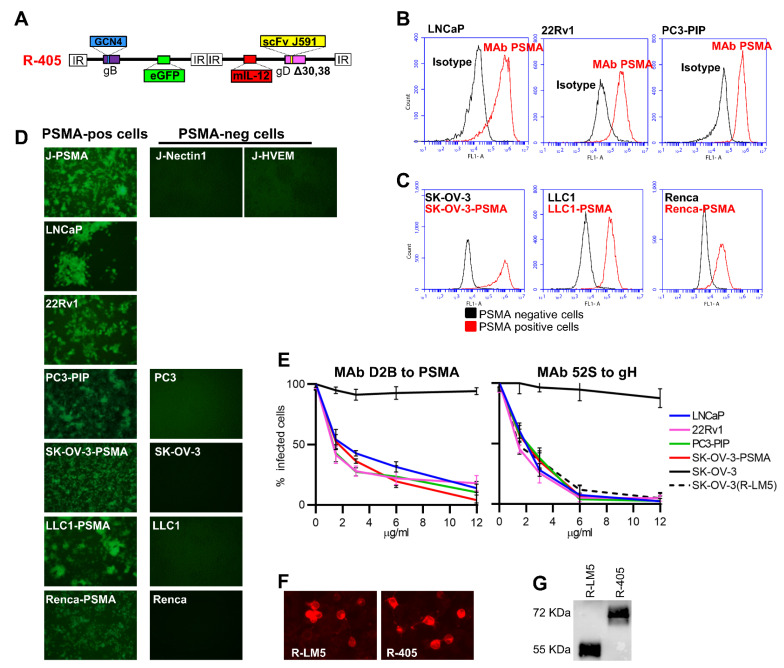
Tropism of R-405 for PSMA-positive cells. (**A**) Genome organization of R-405. The drawing shows the linear map of R-405 genome. Glycoprotein gB gene carries the insertion of GCN4 peptide [45]. The single peptide murine IL-12 is inserted in the US1-US2 intergenic locus. The gD gene carries the deletion of amino acid 30 and the replacement of amino acid 38 with scFv to PSMA. EGFP (enhanced green fluorescence protein) is inserted in the UL37-UL38 locus. (**B**). Flow cytometry quantification of PSMA in LNCaP, 22Rv1 and in PC3-PIP cells. Samples were reacted with MAb D2B to PSMA (red lines) or isotype control (black lines), washed twice, then reacted with the secondary antibody Alexa Fluor™ 488 anti-mouse IgG. (**C**) Flow cytometry quantification of transgenic PSMAΔ in SK-OV-3-PSMA, LLC1-PSMA and Renca-PSMA cells (red lines, details above). The corresponding wt cells were included as control (black lines). (**D**) Infection of the indicated PSMA-positive cells and lack of infection of PSMA-negative cells by R-405. Infection was detected by EGFP fluorescence. Magnification 100×. (**E**) Inhibition of R-405 infection by MAb D2B to PSMA (left panel) and by neutralizing MAb 52S to HSV gH (right panel). Dotted line in the right panel: SK-OV-3 cells infected with wt R-LM5. Each column is the average of triplicate samples ± standard deviation (SD). (**F**) Detection of gD protein in the SK-OV-3-PSMA cells infected with R-LM5 or R-405 viruses by indirect immunofluorescence. SK-OV-3-PSMA were infected with the indicated virus (0.01 PFU/cell); 24 h later, cells were fixed with methanol (this treatment also quenched virus-encoded EGFP), reacted with anti-gD MAb BD80, and with the secondary antibody anti-rabbit DyLight 549. Magnification 600×. (**G**) Efficiency of gD incorporation in R-405 virions. Purified R-LM5 (lane 1) or R-405 (lane 2) virions were subjected to SDS-PAGE, transferred to nitrocellulose membranes, and visualized by Western blotting with the conformation-independent MAb BD80, directed against C-terminus of gD ectodomain (264–274 aa). gD from R-405 contained the scFv to PSMA and exhibited a lower electrophoretic mobility than wt-gD present in the R-LM5 virus. On the left, the predicted molecular weights of the wt-gD (55 KDa) and scFv(PSMA)-gD (72 KDa) are reported.

We engineered a novel PSMA-retargeted oHSV, called R-405, the backbone of which was similar to that of R-337, the prototype of third generation retargeted oHSVs [24]. R-405 genome is depicted in Figure 1A. The key features are: (i) the insertion of a single chain antibody (scFv) to human PSMA in the glycoprotein gD, along with the deletion of gD aminoacidic (aa) residues 30 and 38; these modifications detarget the virus tropism from natural receptors Nectin1 and herpes virus entry mediator (HVEM) and retarget it to PSMA; (ii) the insertion of the GCN4 peptide in gB for manufacturing purposes; specifically, the GCN4 peptide in gB interacts with an artificial cognate receptor expressed in ad hoc-designed producer cell lines [19,24,45]; the insertion of a single peptide form of murine IL-12 for arming; the single peptide IL-12 consists of the p40 subunit (IL12B) linked to the p35 subunit (IL12A) by means of a 15 amino acid long Glycine-Serine linker; it is produced at higher amounts than the hetero-dimeric IL-12, and causes no adverse effects when expressed intratumorally [24,46,47]. 

To verify whether R-405 could cause a bona fide infection of PSMA-positive human prostate tumor cells, PSMA-positive LNCaP cells derived from human androgen-dependent prostate adenocarcinoma lymph node metastasis [48], and PSMA-positive 22Rv1 cells, a human prostate carcinoma epithelial cell line [49] were selected. PC3 cells derived from human PSMA-negative prostate cancer metastasis [50] and PC3-PIP cells which transgenically express PSMA were included. The extent of PSMA expression in the human PC cell lines is shown in Figure 1B.

To express human PSMA in additional human and murine tumor cells, a form of PSMA deleted of the endocytosis motif located in the N-terminal cytoplasmic tail (PSMAΔ) was engineered to maximize and stabilize its cell surface expression. The PSMAΔ was expressed in SK-OV-3 cells, a human tumor cell line routinely utilized in our laboratory for the high titer production of HSV stocks, and in the murine tumor LLC1 and Renca cells, syngeneic with C57BL/6 and BALB/c mice, respectively, thus generating LLC1-PSMAΔ and Renca-PSMAΔ (hereinafter called LLC1-PSMA and Renca-PSMA). The extent of PSMAΔ expression is shown in Figure 1C. We previously reported on the construction of the J-PSMA cell line [37]. The parental J cells do not carry a receptor for HSV and cannot be infected by HSV unless they transgenically express Nectin1 or HVEM [36]. Figure 1D shows the specific tropism of R-405 for cells positive for human PSMA. R-405 infected J-PSMA and failed to infect J-Nectin1 or J-HVEM cells; the recombinant virus infected SK-OV-3-PSMA cells, LLC1-PSMA and Renca-PSMA, but not the corresponding wild type PSMA-negative SK-OV-3, LLC1 and Renca cells. Figure 1D additionally shows that R-405 infected the PSMA-positive LNCaP, 22Rv1 and PC3-PIP cells with high efficiency, but failed to infect the PSMA-negative PC3 cells. Altogether the appropriate PSMA-directed tropism was confirmed in all the cell systems utilized, along with the infection of authentic human PSMA-positive prostate cancer cells. It was ascertained whether R-405 infection took place through PSMA by checking whether MAb D2B to PSMA blocked infection [39]. As a positive control, the neutralizing MAb 52S to glycoprotein H (gH) was included [40]. Figure 1E shows that MAb to human PSMA reduced R-405 infection in a dose-dependent manner in human prostate cancer LNCaP and 22Rv1 cells as well as in PC3-PIP and SK-OV-PSMA cells. Conversely, MAb 52S inhibited infection in all cell lines; MAb 52S also inhibited infection of wt SK-OV-3 cells with wt R-LM5. To verify that the chimeric scFv(PSMA)-gD encoded by R-405 is not defective in cell expression and in incorporation in virions, the cell expression of scFv(PSMA)-gD to that of wt-gD encoded by the wt virus R-LM5 was compared using indirect immunofluorescence of infected SK-OV-3-PSMA cells (Figure 1F). R-405 and R-LM5 virions were purified and it was ascertained that they contained comparable amounts of scFv(PSMA)-gD or wt-gD using western blot with the conformation-independent MAb BD80 against the C-terminus of gD ectodomain (aa 264–274, a region of gD which was not modified in R-405). Figure 1F,G show that there was no major difference between scFv(PSMA)-gD and wt-gD in the extent of cell expression and incorporation in virions.

### 3.2. Infectivity, Cell-to-Cell Spread, and Cytotoxicity Caused by R-405 in Different Cell Lines

**Figure 2:**
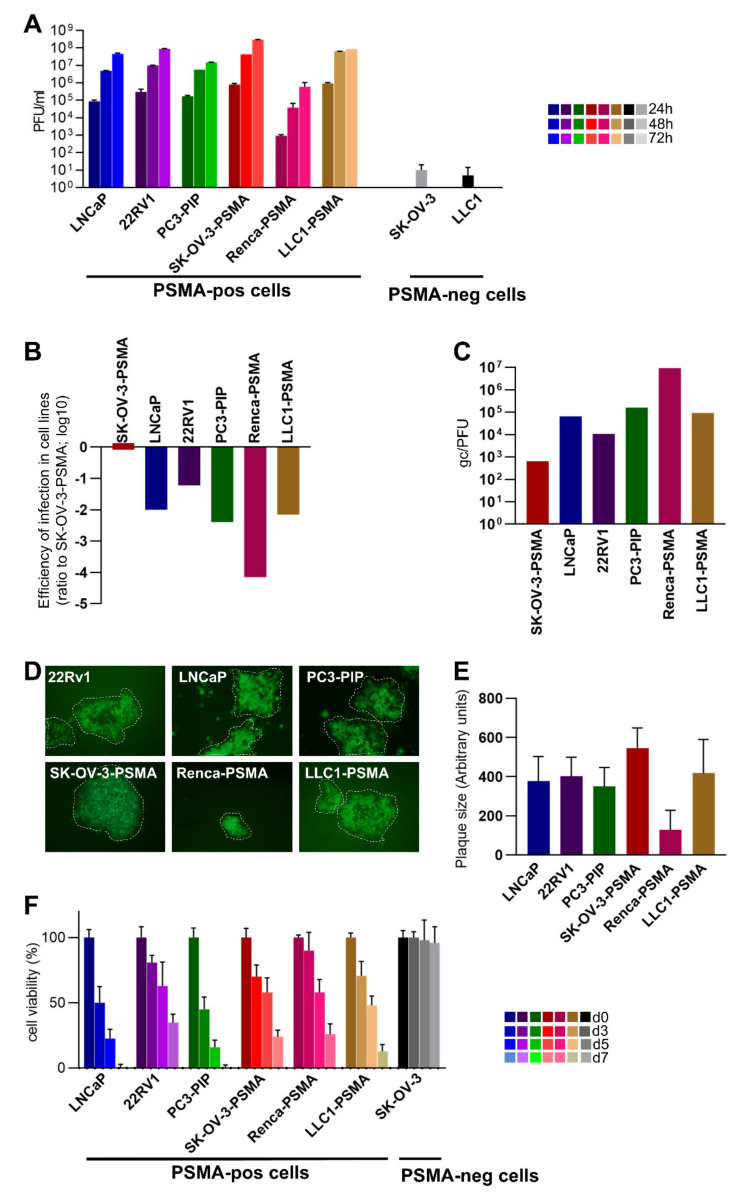
Infectivity, cell-to-cell spread, and cytotoxicity caused by R-405 in different PSMA-positive cell lines. (**A**) Time course of R-405 replication in the indicated cells lines. Infected cells were harvested at 24, 48, and 72 h after infection at 0.1 PFU/cell, as titrated in the respective cell. Progeny virus was titrated in SK-OV-3-PSMA cells. Each column is the average of triplicate samples ± SD. (**B**) Efficiency of R-405 infection. Replicate aliquots of R-405 were plated onto SK-OV-3-PSMA, LNCaP, 22RV1, PC3-PIP, Renca-PSMA, and LLC1-PSMA cells. The number of plaques formed in the indicated cell lines is reported as the ratio to the number of plaques formed in SK-OV-3-PSMA cells. (**C**) Genome copies per PFU values were determined as described [42]. (**D**) Representative R-405 plaques in the indicated cell lines. (**E**) Average plaque size of R-405. For each of the indicated cell lines, six pictures were taken, plaque areas were measured by Nis Elements-Imaging software (Nikon) and plotted ± SD. Magnification 100×. (**F**) Time course of cytotoxic effect of R-405 on the indicated cell lines. Cytotoxicity was determined by AlamarBlue as detailed [21] and expressed at each time point as the percentage of infected relative to uninfected cells. Each column is the average of triplicate samples ± SD.

The extent of R-405 replication in the above cells was quantified in a virus yield experiment at 24, 48, and 72 h after infection. Infection was carried out at 0.1 plaque-forming unit (PFU)/cell, according to the R-405 titer in each cell line (Figure 2A). R-405 replicated in LNCaP and 22Rv1 cells, strengthening the notion that R-405 successfully targeted PSMA-positive human prostate cancer cells; R-405 also replicated in PC3-PIP cells. The yields in LNCaP and 22Rv1 cells were similar. The replication in SK-OV-3-PSMA cells, but not in the wt SK-OV-3 cells additionally underscored the fact that R-405 needed PSMA for infection and replication. Of the two PSMA-transgenic murine cell lines, R-405 replicated at high yield in LLC1-PSMA cells, at lower yield in Renca-PSMA cells and failed to replicate in wt LLC1 cells (Figure 1D and Figure 2A). To additionally quantify the differences in the ability of R-405 to infect the above cell lines, replicate amounts of R-405 were plated and the resulting number of plaques were scored. Plating efficiency in Figure 2B shows the decrease in the number of plaques relative to those formed in SK-OV-3-PSMA cells. For each cell line, Figure 2C shows the number of genome copies (gc) required to give rise to a PFU in the indicated cell lines. Parenthetically, the gc/PFU ratio of R-405 in SK-OV-3-PSMA cells (648 gc/PFU) is a figure compatible with those exhibited by retargeted oHSVs [42], and approximately three/four-fold higher than that exhibited by wt-HSV. The gc/PFU values of R-405 in LNCaP, 22Rv1, PC3-PIP e LLC1-PSMA cell lines were 2 orders of magnitude higher than those in SK-OV-3-PSMA and, in Renca-PSMA, the values were 4 orders of magnitude higher. Cumulatively, Figure 2B,C confirmed that the human PC cell lines and the LLC1-PSMA were infected with R-405 at a similar efficiency whereas the infection of Renca-PSMA was 2 orders of magnitude less efficient. R-405 cell-to-cell spread was determined by measuring the plaque size in the PSMA-positive cell lines and was similar for all the cells tested, except for the Renca-PSMA cells, as expected. The ability to form plaques denoted that the R-405 progeny virus had the ability to spread to adjacent PSMA-positive cancer cells. It predicted that, once R-405 was administered to tumors, the progeny virus could spread to adjacent tumor cells within the treated tumors, (Figure 2D,E). Finally, whether R-405 caused the death of PSMA-positive cells was assessed. The cells indicated were infected with R-405, and cytotoxicity was quantified using the AlamarBlue test at 3, 5, and 7 d after infection. Figure 2F shows that R-405 was cytotoxic to all PSMA-positive cells. Altogether R-405 fulfilled several criteria which concordantly indicated that it infected exclusively PSMA-positive cells, replicated in them and was cytotoxic. Importantly, R-405 infected, replicated in, and was cytotoxic to human PSMA-positive prostate cancers, and spared human PSMA-negative prostate cancer cells. We concluded that R-405 was suitable for specifically targeting human PSMA-positive PC cells. As far as the murine tumor cells were concerned, they differed significantly one from the other; the LLC1-PSMA expressed PSMA at a higher level (and at a level similar to that in LNCaP and 22Rv1 cells) and sustained R-405 infection at much higher efficiency than Renca-PSMA. With respect to the level of PSMA expression, LLC1-PSMA and Renca-PSMA can be considered to mirror PSMA_HIGH_ and PSMA_LOW_ human PC cells, respectively [33].

### 3.3. In Vivo Efficacy of R-405 Monotherapy against LLC1-PSMA Tumors

To provide the first proof-of-principle evidence that R-405 exerts antitumor efficacy against PSMA-positive tumors in syngeneic murine models, we made use of the LLC1-PSMA and Renca-PSMA tumor cells described above, syngeneic with C57BL/6 and BALB/c mice, respectively. Two systems were utilized since it has been well established that tumors vary greatly in their mechanisms of immune response and evasion, also called immune editing. On one extreme are tumors which are very poor in eliciting the immune response; they are designated as immunologically cold, or, in more severe examples, immunologically desert. At the other extreme are tumors which are competent in eliciting an immune response (immune competent or immunologically warm/hot) which they dampen by a variety of immune suppressive mechanisms [51,52,53]. In an accompanying paper in the current Special Issue [54], we reported on the molecular profiling of the immunologically cold LLC1 and the immunologically warm CT26 tumors and the effects of the HER2-retargeted R-337. With respect to the immune evasion strategies, Renca cells are similar to CT26 cells [51]. In addition, properties shown in Figure 1 and Figure 2 indicate that, with respect to the level of PSMA expression, LLC1-PSMA and Renca-PSMA can be considered as mirroring PSMA_HIGH_ and PSMA_LOW_ human PC cells, respectively.

**Figure 3:**
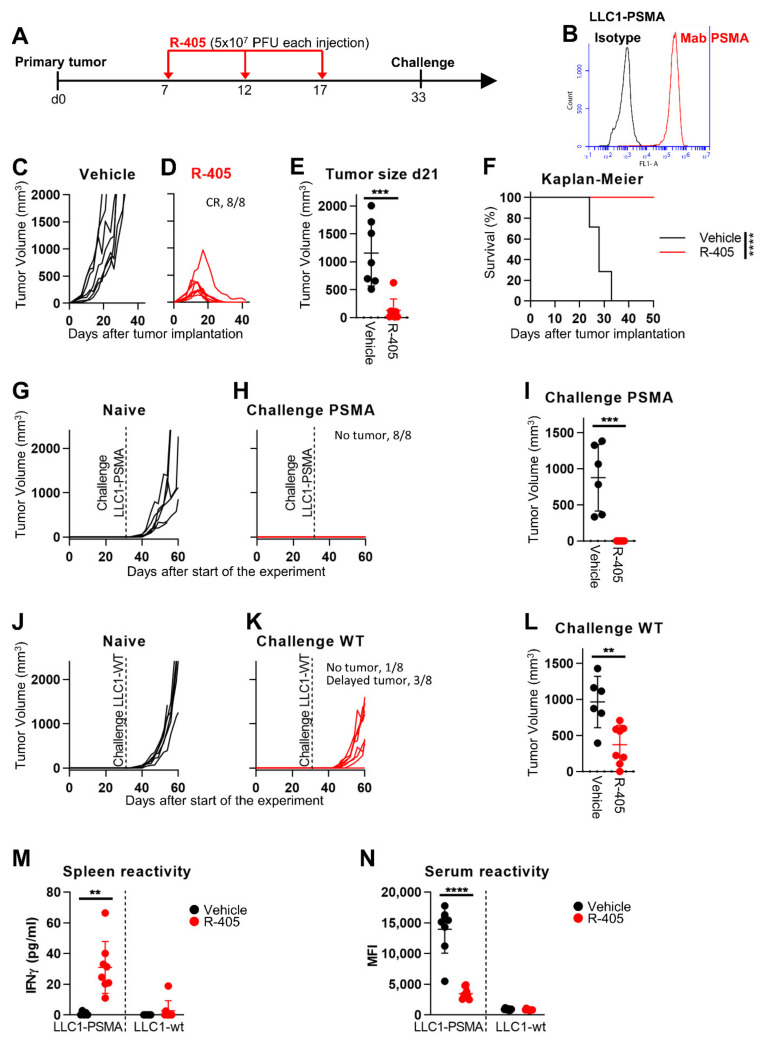
Efficacy of R-405 monotherapy on the growth of LLC1-PSMA tumors. (**A**) Schedule of treatments. Six-to-eight weeks old C57BL/6 mice were s.c. implanted in the left flank with 1 × 10^6^ LLC1-PSMA cells in 100 μL of PBS, according to [24]. 7 d later, when the tumor volumes averaged 70–100 mm^3^, mice received 3 intratumoral injections of R-405 (5 × 10^7^ PFUs, diluted in 50 μL PBS) or vehicle (50 μL PBS), at 5 days intervals. At d 33, the R-405-treated mice, which had survived the primary tumor received simultaneously two contralateral challenge tumors made of LLC1-PSMA and LLC1-wt cells (1 × 10^6^/mouse), respectively. (**B**) Flow cytometry quantification of PSMA in LLC1-PSMA tumors. Single cell suspensions were prepared from freshly isolated LLC1-PSMA tumors at sacrifice and plated. Three days after, non-adherent cells were removed; samples were trypsinized and reacted with MAb D2B to PSMA (red line) or with the isogenic control (black line), washed twice, then reacted with the secondary antibody Alexa Fluor™ 488 anti-mouse IgG. (**C**–**E**) Kinetics of tumor growth in mice treated with vehicle (**C**) or R-405 (**D**). The numbers reported in each panel indicate the numbers of mice which were completely cured from tumors (complete response, CR), or which showed a delay/reduction in tumor growth (partial response, PR). The mice were scored PR when the tumor volume was <50% smaller than the mean size of the tumors in the vehicle group, in at least 2 consecutive measurements. (**E**) Volumes of the primary tumors at d 21 after implantation. (**F**) Kaplan-Meier survival curves of the two groups of mice. (**G**–**K**) Kinetics of growth of challenge tumors in naïve mice (**G**,**J**), or in the R-405 survivors’ arm (**H**,**K**). Mice received simultaneously LLC1-wt (**J**,**K**) and LLC1-PSMA cells (**G**,**H**) in the right and left flanks, respectively. No tumor growth and delayed tumor growth are indicated. (**I**,**L**) Volumes of challenge tumors. LLC1-PSMA (**I**); LLC1-wt (**L**). (**M**,**N**) Long-term T and B cell immunity to LLC1-PSMA and LLC1-wt cells. (**M**) Splenocyte harvested at sacrifice were incubate with LLC1-PSMA or LLC1-wt cells. Reactivity was quantified as IFNγ release. (**N**) Antibodies to LLC1-PSMA or LLC1-wt cells in sera harvested at sacrifice. Reactivity was measured in CELISA test. (**E**,**I**,**L**–**N**) Circles correspond to individual mice, horizontal line indicates the mean value, and vertical bars ± SD. (**E**,**F**,**I**,**L**–**N**) Statistical significance was calculated by the *t*-test (**E**,**I**,**L**–**N**) or by Log-rank (Mantel-Cox) test (**F**) and expressed as ** = *p*-value < 0.01; *** = *p*-value < 0.001; **** = *p*-value < 0.0001. Color code: mice treated with vehicle or R-337 are indicated in black or red, respectively.

In the first series of in vivo experiments, LLC1-PSMA tumor cells were implanted subcutaneously (s.c.) in syngeneic C57BL/6 mice. The experimental design is shown in Figure 3A. Briefly, the mice received 3 intratumoral (i.t.) injections at 5 d time interval, 5 × 10^7^ PFUs for each dose. This dose was chosen based on our previous study regarding the efficacy of the HER2-retargeted oHSV called R-337 against HER2-positive LLC1 tumors [24]. Ex vivo cultures of LLC1-PSMA tumors showed high expression levels of the PSMA transgene (compare Figure 1C to Figure 3B). Figure 3C,D shows that R-405 administration to established LLC1-PSMA tumors caused a complete inhibition of tumor growth and subsequent tumor regression (complete response, CR, in 100% of the mice). Figure 3E shows the highly significant difference in tumor size at d 21 between untreated and R-405-treated mice. The Kaplan-Meyer survival curve mirrored this huge difference (Figure 3F).

Subsequently, all the R-405-treated mice were simultaneously implanted with two challenge tumors in the right and left flanks, made up of LLC1-PSMA or wt LLC1 (herein LLC1-wt) cells, respectively. Figure 3G–I shows that the mice were fully protected from the LLC1-PSMA challenge. Surprisingly, they were also partly protected from LLC1-wt challenge (Figure 3J–L), implying that R405 treatment resulted in immune response not only to PSMA but also to LLC1 tumor antigens. In fact, R-405 elicited a long-term T-cell response seen in the splenocytes harvested at sacrifice and measured as an Interferon gamma (IFNγ) release when co-cultured with LLC1 cells. Figure 3M documents the splenocyte reactivity to LLC1-PSMA cells and, to a lesser extent, to LLC1-wt cells. With respect to antibody response, the tumor-bearing mice exhibited reactivity to LLC1-PSMA but not to LLC1-wt cells, consistent with the notion that mice were not tolerant to human PSMA. Surprisingly, the antibody reactivity to PSMA-positive cells was lower in the R-405-treated mice (Figure 3N). Whether this reflected a lower stimulation of the B-cell compartment consequent to the reduction in tumor volume, peculiar B-T cell circuits, or other mechanisms remains to be investigated. The distant protection and concomitant immune responses induced by the tumor treatment with R-405 exemplified an in situ vaccination, a phenomenon observed with OVs [55,56,57].

### 3.4. Changes to the LLC1-PSMA Tumor Microenvironment Induced by R-405

**Figure 4:**
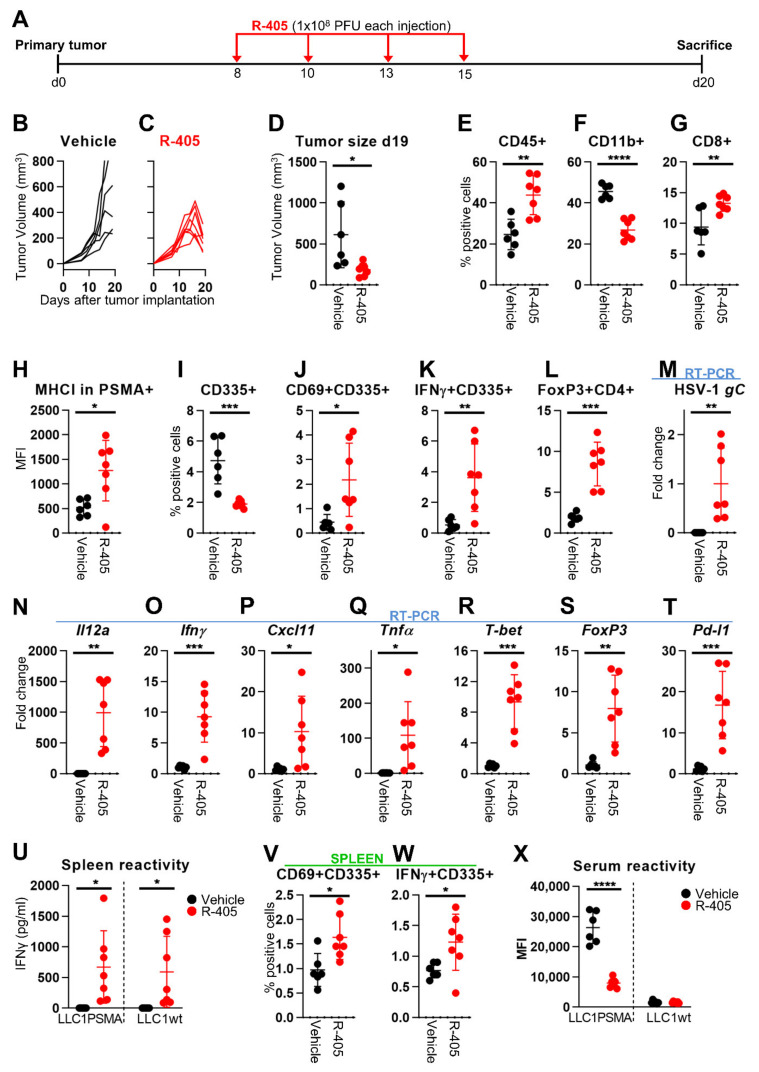
Immune modifications to TME and long-term T and B responses induced by intratumoral R-405 monotherapy. (**A**). Schedule of treatment. (**B**,**C**) Kinetics of tumor growth in C57BL/6 mice treated with vehicle (**B**) or R-405 (**C**). (**D**) Tumor volumes at d 19. (**E**–**L**) Immune cell populations in tumors. Single cell suspensions were prepared from freshly isolated LLC1-PSMA tumors at sacrifice. For each sample, 2 × 10^6^ cells were blocked with α-CD16/32 Ab, and then reacted with the antibodies CD8a-PE, CD45-PE-Cy7, CD335-PE, FoxP3-PE, CD11b-FITC, CD69-PercP, MHC-I(H-2Kb)-APC, PD-L1-APC, and IFNγ-APC. Data were acquired by means of BD C6 Accuri. CD8 (CD8+ cells), NK (CD335+ cells) and myeloid cells (CD11b+ cells) were gated on CD45+ subpopulation. Activated (CD69+ of IFNγ+) NK cells were gated on CD335+ subpopulation. Tregs (FoxP3+ CD4+) were gated on CD4+ population. MHC-I levels were assayed in cell population gated on PSMA+. (**M**–**T**) Expression profile of HSV-1 marker, immune related markers, and immune-related transcription factors. Tumor homogenates were employed for total RNA purification and 1.2 µg of RNA was employed for the cDNA synthesis. cDNAs were assayed by real-time PCR with Taqman probes. The levels of expression were determined using the ΔΔCt method, normalized on the *Rpl13a* housekeeping gene and on the mean of the vehicle-treated group. For panel M, HSV-1 *gC* values were normalized on the mean of the R-405-treated group. (**U**) Immune response in splenocytes to LLC1-PSMA and to LLC1-wt cells was quantified as IFNγ secretion in the culture medium. (**V**,**W**) Splenocyte reactivity and immune cell populations in spleens. Sample preparation and staining as described for tumors. (**X**) Serum antibody reactivity to LLC1-PSMA and to LLC1-wt cells. (**D**–**X**) Circles correspond to individual mice, horizontal line indicates the mean value, and vertical bars ± SD. (**D**–**X**) Statistical significance was calculated by the *t*-test and expressed as * = *p*-value < 0.05; ** = *p*-value < 0.01; *** = *p*-value < 0.001; **** = *p*-value < 0.0001. Color code: mice treated with vehicle or R-337 are indicated in black or red, respectively.

Collectively, the above data indicated that R-405 monotherapy served as a potent immunotherapeutic treatment against LLC1-based tumors. The purpose of the subsequent experiments was to analyze in some detail the immune response elicited by R-405. The experiment in Figure 3 was replicated (Figure 4A); the mice were sacrificed 5 d after the end of the virus treatment when the tumor growth curve in the treated mice was declining (Figure 4B–D). Three series of analyses were conducted, namely characterization of tumor-infiltrating lymphocytes, of intratumoral immune mediators, and of systemic T response and serum antibodies. With respect to infiltrating lymphocytes, R-405-treated tumors exhibited an overall increase in leucocytes (CD45+), including CD8+ cells, and a decrease in the myeloid (CD45+ CD11b+) subpopulation (Figure 4E–G). The natural killer (NK) cells were decreased in the tumors treated (Figure 4I), consistent with the increase in MHC-I expression in PSMA-positive tumor cells. However, the fraction of activated NK cells (CD69+, IFNγ+) was increased (Figure 4H–K); hence, the reduction of NK population in treated tumors could be also the result of activation-induced cell death. The T regulatory cells detected as a CD4+ FoxP3+ population were increased (Figure 4L), in agreement with previous findings with LLC1-HER2 tumors treated with retargeted-oHSVs [20,24]. A compensatory increase in FoxP3-positive cells during T-cell activation was observed with other OVs, e.g., Newcastle disease virus [58]. A global view of the TME was carried out using RT-PCR analysis which highlighted the high-level expression of the R-405 marker *gC* glycoprotein and of the R-405-encoded *Il12a* (Figure 4M,N). The intratumoral mediators of innate response *Ifnγ*, *Cxcl11*, and tumor necrosis factor alpha (*Tnfα*) were increased as was the *T-bet* transcription factor, which favored Th1 polarization, and the T-regulatory cell *FoxP3* factor (Figure 4O–S). Of note, R-405 treatment increased *Pd-l1* expression in the tumor cell population (Figure 4T), raising the possibility that R-405 sensitizes to ICIs. Evidence for long-term adaptive T response rested on high splenocyte reactivity to LLC1-PSMA and LLC1-wt cells in agreement with protection from PSMA positive and from LLC1-wt challenge tumors (Figure 4U). Moreover, an increase in the CD69 and IFNγ markers in the splenic NK cell population, a feature similar to that in tumor infiltrating immune populations was observed (Figure 4V,W). Consistent with the decrease in serum reactivity described above (see Figure 3N), a decrease in serum antibodies to LLC1-PSMA was observed (Figure 4X).

### 3.5. R-405 and Anti-PD-1 Combination Therapy against LLC1-PSMA Tumors

**Figure 5:**
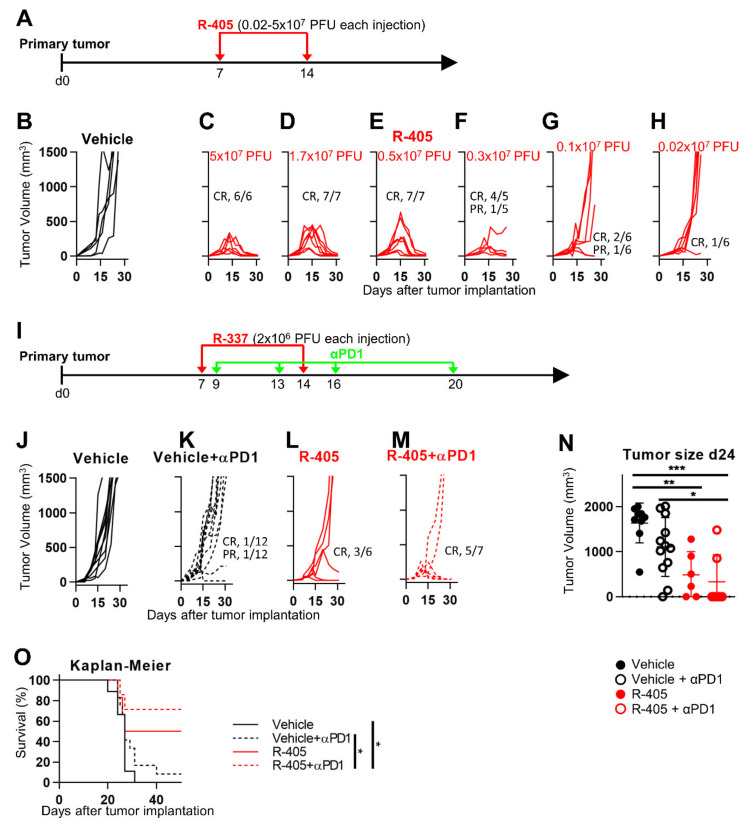
Therapeutic effects of R-405 in combination with anti-PD-1 antibody on the growth of LLC1-PSMA tumors. (**A**) Schedule of the monotherapy treatments. Mice were implanted with LLC1-PSMA cells. At d 7 after implantation, when tumors reached the average volume of 70–100 mm^3^, mice received 2 i.t. injections of R-405 at indicated dosages ranging from 5 × 10^7^ to 2 × 10^5^ PFUs or vehicle at 7 d time interval. (**B**–**H**) Tumor growth curves at indicated dosages. (**I**) Schedule of the combination treatments. Mice received two i.t. injections of R-405 at 7 d interval and 4 i.p. injections of anti-PD-1 at 3–4 d intervals. (**J**–**M**) Tumor growth curves in mice receiving the monotherapies or combination treatment. Figures in panels indicate the number of mice exhibiting complete response (CR) or partial response (PR). (**N**) Volumes of the primary tumors at d 24 after implantation. Circles correspond to individual mice, horizontal line indicates the mean value, and vertical bars ± SD. (**O**) Kaplan-Meier survival curves of the four indicated groups of mice. (**N**,**O**) Statistical significance was calculated by means of the ANOVA test with Tukey’s correction (**N**) or Log-rank (Mantel-Cox) test with Bonferroni’s correction (**O**), and expressed as * = *p*-value < 0.05; ** = *p*-value < 0.01; *** = *p*-value < 0.001. Color codes: mice treated with vehicle (black) or R-337 (red). Full circles and continuous lines, monotherapies. Open circles and dotted lines, combination therapies.

We subsequently asked whether R-405 was sensitized to anti-PD-1 therapy. This experiment required a dose of R-405, which protected approximately 50% of the mice, and not 100% of the mice, as was the case in the experiment in Figure 3. To this end, a decreasing dose experiment was carried out. Mice carrying established LLC1-PSMA tumors were treated twice with doses of R-405 lower than those utilized in Figure 3, ranging from 5 × 10^7^ to 2 × 10^5^ PFUs (Figure 5A). The dose/response curves are illustrated in Figure 5B–H. The three highest dosages (down to 5 × 10^6^ PFUs) resulted in a CR in 100% of the mice. The lowest dosage 2 × 10^5^ PFUs protected only 1 out of six mice. The 3 × 10^6^ and 1 × 10^6^ PFU dosages induced a CR in 80% and 33% of the mice, respectively.

The schedule of subsequent combination experiments is shown in Figure 5I. Anti-PD-1 antibodies at the dosage utilized herein as monotherapy induced a CR in 1 out of 12 mice (Figure 5J,K). R-405 was administered twice at the 2 × 10^6^ PFU dosage for each injection; it induced a CR in 50% of the mice (Figure 5L). The combined treatment induced a CR in 5/7 (71%) of the mice (Figure 5M). The tumor volumes at d 24 and the Kaplan-Meier survival curves showed highly statistically differences in the combination relative to the anti-PD-1 monotherapy or vehicle (Figure 5N,O). Taken altogether, the results argue in favor of an additive effect.

### 3.6. R-405 Monotherapy and Combination Therapy against Renca-PSMA Tumors

**Figure 6:**
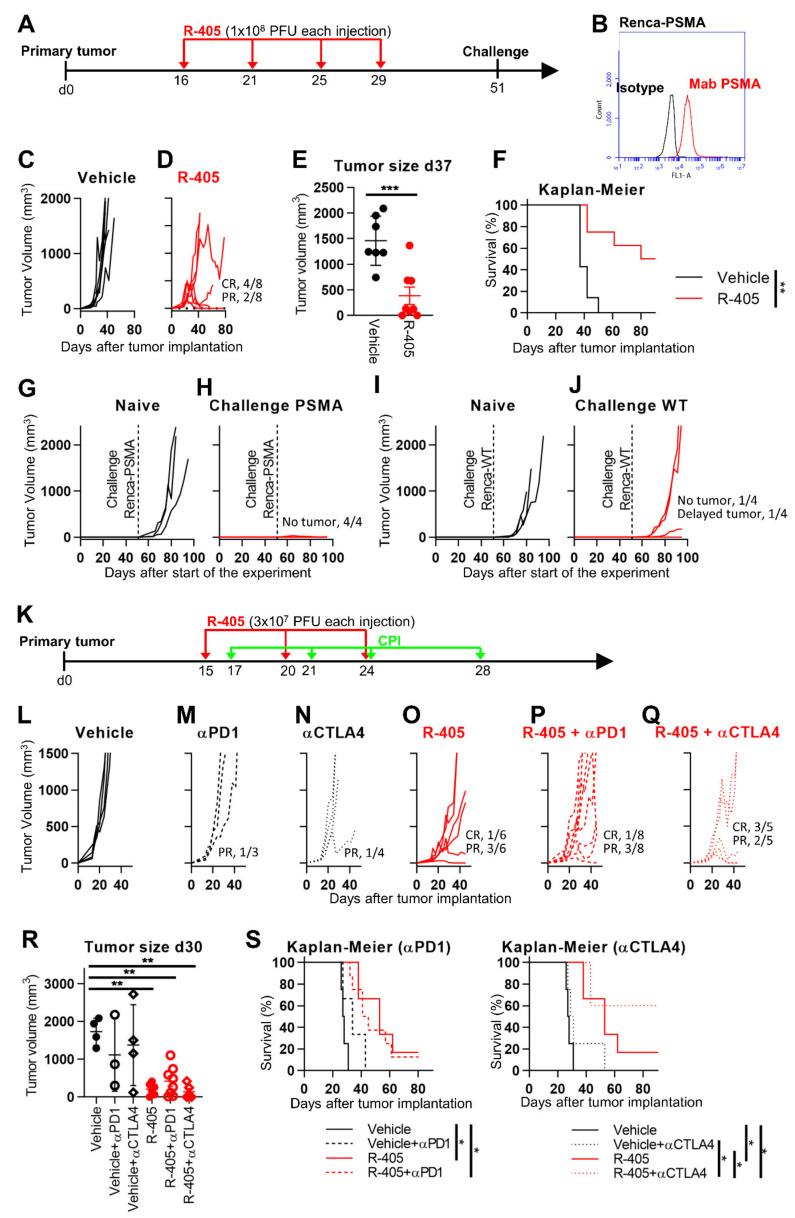
Efficacy of R-405 monotherapy and combination therapies on the growth of Renca-PSMA tumors. (**A**) Schedule of monotherapy treatment. Mice were s.c. implanted in the left flank with 1 × 10^6^ Renca-PSMA cells in 100 μL of PBS. 16 d later, when the tumor volumes averaged 70–100 mm^3^, mice received 4 intratumoral injections of R-405, 1 × 10^8^ PFUs each, diluted in 50 μL PBS or vehicle (50 μL PBS), at 4-5 days intervals. At d 51, the R-405-treated mice that survived the primary tumor received simultaneously two contralateral challenge tumors made of Renca-PSMA and Renca-wt cells (1 × 10^6^/mouse), respectively. (**B**) Flow cytometry quantification of PSMA in Renca-PSMA tumors. Samples were prepared as detailed in Figure 3. (**C**,**D**) Kinetics of tumor growth in mice treated with vehicle (**C**), or R-405 (**D**). The numbers reported in each panel indicate the numbers of mice which exhibited C.R. or P.R. (**E**) Volumes of the primary tumors at d 37 after implantation. (**F**) Kaplan-Meier survival curves of the two groups of mice. (**G**–**J**). Tumor growth curves in mice implanted with challenge tumors made of Renca-PSMA (**G**,**H**) or Renca-wt cells (**I**,**J**). (**K**) Schedule of combination treatments. Mice received 3 i.t. injections of R-405 at 4–5 d interval and 3 i.p. injections of the ICI at the indicated time interval. (**L**–**Q**) tumor growth curves in mice receiving vehicle, the indicated monotherapies or combination therapies. (**R**) Tumor volumes at d 30 in the six groups of mice. (**S**) Kaplan-Meier survival curves of the indicated groups of mice. (**E**,**R**) Circles correspond to individual mice, horizontal line indicates the mean value, and vertical bars ± SD. (**E**,**F**,**R**,**S**) Statistical significance was calculated by means of the ANOVA test with Tukey’s correction (**R**), Log-rank (Mantel-Cox) test (**F**), Log-rank (Mantel-Cox) test with Bonferroni’s correction (**S**), or *t*-test (**E**), and expressed as * = *p*-value < 0.05; ** = *p*-value < 0.01; *** = *p*-value < 0.001. Color codes: mice treated with vehicle (black) or R-337 (red). Full circles and continuous lines, monotherapies. Open circles and dashed lines, combination therapies with anti-PD-1; open diamonds and dotted lines, combination therapies with anti-CTLA4.

As an immunologically warm model, Renca-PSMA cells implanted s.c. and treated i.t. with R-405 were utilized (Figure 6A). Upon implantation in mice and tumor growth, the expression of PSMA was maintained in in vivo-grown Renca-PSMA tumors (compare Figure 6B to Figure 1C), which grew at a somehow slower rate than the untreated LLC1-PSMA tumors (compare Figure 3C with Figure 6C). Figure 6D shows that four doses of 1 × 10^8^ PFUs each resulted in a CR in 50% of the mice and a partial response (R) in 25% of the mice. The reduction in tumor volume at d 37 and the Kaplan-Meier survival curve showed highly significant differences between the untreated and R-405-treated mice (Figure 6E,F). The lower efficacy relative to that seen with LLC1-PSMA tumors may be consequent to the low-level expression of PSMA in Renca-PSMA relative to LLC1-PSMA (see Figure 1C, Figure 3B, and Figure 6B), in the extent of R-405 replication and cell-spread seen in the two cell types, and possibly of fewer changes to the TME due to lower viral replication. Notably, the highest yield reached in Renca-PSMA and in LLC1-PSMA differed by two orders of magnitude (6 × 10^5^ PFU/mL and 8.6 × 10^7^ PFU/mL at 72 h, respectively).

The mice which survived the primary tumor were simultaneously implanted with two s.c. challenge tumors made up of Renca-PSMA and Renca-wt cells. The extent of distant protection is reported in Figure 6G–J. Briefly, the mice were fully protected from Renca-PSMA (Figure 6G,H), and poorly protected from Renca-wt tumor cells (Figure 6I,J), indicating that, in the Renca-PSMA model, long-term protection was strong against PSMA-positive tumors, but weak against the Renca-wt cells.

In the final experiment (Figure 6K), the effects of anti-PD-1 or anti-CTLA4 antibody monotherapies, and the respective combination therapies (Figure 6L–Q) were evaluated. Renca tumors are known to be partially sensitive to the anti-CTLA4 monotherapy [59,60]. Monotherapies with the antibodies exerted no-to-modest effects (Figure 6M,N). At the dosage utilized, R-405 exerted a CR and a PR in 1/6 and 3/6 mice, respectively. The effect of the combination therapy was higher with anti-CTLA4 than with anti-PD-1, and cumulatively affected 100% of the mice (3/5 CR and 2/5 PR), highlighting that the response needed to be determined as a function of tumor specificities. The tumor volumes at d 30 in the six groups of animals is reported in Figure 6R. The difference was highly statistically significant between the untreated mice and those receiving the R-405 monotherapy or the combinations with ICIs. The Kaplan-Meier curve showed a statistically significant advantage for mice receiving the R-405 (monotherapy or combination) relative to the vehicle. Notably, the combined R-405 and anti-CTLA4 therapy improved the survival of mice with respect to the R-405 and anti-CTLA4 monotherapies (Figure 6S), while R-405 and anti-PD1 combination did not show any advantage relative to R-405 monotherapy.

## 4. Discussion

We report on a first proof-of-principle study of the properties and in vivo efficacy of a fully virulent oHSV retargeted to PMSA as an example of precision medicine and in situ vaccination approach against PC. The key features were as follows. (i) The PSMA-retargeted IL-12-armed oHSV, called R-405, infected exclusively PSMA-positive cells and spared PSMA-negative cancerous or non-cancerous cells. It infected the cells and caused cytopathic death of the PSMA-positive human prostate cancer LNCaP and 22Rv1 cells, spared the PSMA-negative human prostate cancer PC3 cells, but infected the PC3-PIP cells transgenically expressing PSMA. The PSMA served as the port of entry of the R-405 into such cells. Thus, the human PSMA-positive PC cells were specific targets for R-405. With respect to murine tumor cells, the efficacy of the R-405 infection, cell-to-cell spread, and virus-induced cell death were higher in LLC1-PSMA than in Renca-PSMA cells; these features likely resulted from the intrinsic properties of the two murine tumor cells and, possibly, also from the different extent of PSMA expression. (ii) The in vivo key features of R-405 were its high efficacy against primary tumors (in the LLC1-PSMA model, 100% of mice exhibited a CR when treated with high doses of R-405), and the anti-tumor in situ vaccination effect which resulted in protection against distant challenge tumors. Notable, the immune protection was exerted not only towards the PSMA TAA but, in part, also towards the LLC1 tumor antigens, confirming that the retargeted oHSVs primed for antigen-agnostic antitumor immune response. (iii) The bases of the immune protection were the anti-tumor Th1 polarization of the TME (i.e., increased levels of IL-12, IFNγ, interferon-stimulated genes and TNF), the development of systemic tumor-specific T-lymphocytes (splenocytes), infiltration of the tumor masses by CD8+ and activated NK cells, and the inhibition of the mechanisms of tumor tolerance, exemplified by the depletion of tumor-infiltrating myeloid-derived suppressor cells (MDSCs) and the restoration of MHC-I expression in tumor cells. (iv) R-405 sensitized tumors to a checkpoint blockade-based therapy. The virus treatment inflamed the TME through infection and direct oncolysis, activation of innate and adaptive immunity, and expression of pro-inflammatory cytokines (including the virus-encoded IL-12 and the subsequent IFN cascade). Nevertheless, these modifications also triggered some tumor defenses against immune system, including the increase of PD-L1 expression and the tumor infiltration by Tregs. Both effects were caused by higher IFN levels [54,61] and other mechanisms. The combination therapy of R-405 with ICIs likely neutralized these effects: anti-PD-1 could be able to counteract the increase of PD-L1 expression in LLC1-PSMA tumors and prevent the anergy of the anti-tumor T cells, while anti-CTLA-4 could increase T-cell priming against tumor antigens and/or depleting Treg population in Renca-PSMA tumors. (v) R-405 efficacy was exerted towards two different types of tumors, one immunologically cold, and the other immunologically warm; hence, it appeared to be independent of the tumor immune profile and genotype. The high immunotherapeutic efficacy of R-405 was due in part to the IL-12 payload, known to promote the secretion of IFNγ and additional proinflammatory cytokines which, in turn, recruited and activated the immune effector cells. By means of these mechanisms, IL-12 adjuvanted the immunotherapeutic effects of oncolytic viruses [28]. In fact, a number of OVs are armed with IL-12 [28]. The specific form of mIl-12 encoded by R-405 was a single peptide version, which ensured higher levels of cytokine expression within the R-405-treated tumor. R-405 may obviously be improved even more with additional payloads, as appropriate for human PCs. Additional studies should address efficacy in more authentic murine models of PC.

Metastatic castration-resistant prostate cancer remains incurable and fatal, despite the availability of multiple classes of therapy which delay disease progression and prolong life [32]. Beyond the standard of care approaches, the current landscape of treatments against PC includes OV-based treatments, PSMA-targeted therapies, and a personalized vaccine. An overview of these approaches follows:

(i) Previous attempts to generate OVs specific for PC cells have included conditionally replicating transcriptionally retargeted adenoviruses (AdVs) carrying key viral genes under prostate specific antigen (PSA) or PSMA enhancer elements [62,63], an alphavirus encoding PSMA and acting as a vaccine [64], a vesicular stomatitis virus (VSV) pseudotyped with measles virus (MeV) glycoprotein, including a PSMA-retargeted MeV glycoprotein [65], and an MeV genetically retargeted to PSMA by insertion of scFv in the hemagglutinin gene [66]. The latter virus demonstrated in vivo efficacy against xenotransplants of LNCaP and 22Rv1 human PC cells [66]. Its properties against immunocompetent PSMA-positive murine models and the extent of in vivo off-tumor infections were not reported. The PSMA-MeV had not been additionally investigated in the past 10 years. Hence, how effective it is in eliciting anti-tumor in situ vaccination and in sensitizing to ICIs is not known. AdVs and Reolysin are being tested against PC in ongoing phase 1 or 2 clinical trials, without or in combination with ICIs.
(ii) High PSMA expression is frequent (75.21%) in metastatic castration-resistant prostate cancer patients and portends a poor prognosis [31,32,33]. Anti-PSMA antibodies are utilized for diagnostic or combined diagnostic + therapeutic (theranostic) applications, including PSMA-SPECT and the delivery of radiometals. Most of the PSMA-based clinical trials utilize anti-PSMA as a diagnostic or theranostic tool. Of note, a recently closed phase 3 clinical trial regarding the effect of Lutetium-177–PSMA-617 prolonged progression-free survival and overall survival when added to standard care in patients with advanced PSMA-positive metastatic castration-resistant prostate cancer [32]. Notably, a wealth of data convincingly indicated that targeting PSMA results in low toxicity and suggested few off-tumor effects [34,35].
(iii) The importance of PSMA as a target for precision medicine is strengthened by investigations regarding PSMA-targeted chemical antigen receptor T cells (CAR-Ts) [67]. Clinical trials are ongoing. Despite many efforts and rapid developments, the field of CAR-Ts regarding solid tumors still faces difficulties which need to be overcome. Oncolytic viruses, with their ability to attract lymphocytes to TME and to activate them, coupled with high tolerability, could be interesting partners for CAR-Ts.
(iv) Very few trials address anti-PC vaccination. Sipuleucel-T is a dendritic cell (DC)-based vaccine recommended as first-line therapy for the treatment of metastatic castration-resistant prostate cancer [68]. It must be prepared individually for each patient, starting from the patient’s own blood [5,6].

Given the slow progression typical of human PCs, an approach capable of eliciting a long-term adaptive immune response appears to be appropriate. An off-the-shelf therapeutic treatment capable to induce in situ vaccination would respond to this need and would be of practical application and at the reach of a high number of patients. The properties of the PSMA-retargeted oHSVs described in the current proof-of-principle study addressed these challenges. The target patients should include early and advanced stage patients for the purpose of slowing down progression to more advanced stages and to metastatic disease as well as metastatic disease patients. The PSMA-oHSV might ultimately be combined with PSMA T-cell-based therapies to achieve long-term immunoprevention and immunotherapy. We concluded from this proof-of-principle study that PSMA-retargeted oHSVs are worth being additionally investigated as anticancer agents and as candidate therapeutic in situ vaccines against prostate cancer.

## 5. Patents

Herpes Simplex Virus (HSV) with modified tropism, uses and process of preparation thereof-WO2009144755 (divisional patent EP2700405)

Retargeted herpesvirus with a glycoprotein H fusion-WO201612849

Herpesvirus with Modified Glycoprotein B-WO2017211941

Herpesvirus with Modified Glycoprotein D-WO2017211944

Herpesvirus with Modified Glycoprotein H for Propagation in A Cell-WO2017211945
